# Assessment of Motor Performance and Self-Perceived Psychophysical Well-Being in Relation to Body Mass Index in Italian Adolescents

**DOI:** 10.3390/children11091119

**Published:** 2024-09-12

**Authors:** Gaetano Altavilla, Sara Aliberti, Francesca D’Elia

**Affiliations:** 1 Research Centre of Physical Education and Exercise, Pegaso Telematic University, 80143 Napoli, Italy; gaetano.altavilla@unipegaso.it; 2 Department of Human, Philosophical and Education Sciences, University of Salerno, 84084 Fisciano, Italy; saaliberti@unisa.it

**Keywords:** sedentary lifestyle, excess weight, motor skills, self-perception

## Abstract

Background/Objectives: The relationship between psychophysical self-perception (PSP), body mass index (BMI) and motor performance (MP) levels, in quantitative and qualitative terms, can be a very interesting connection to investigate. This study aimed to assess MP and PSP according to gender and weight differences (normal weight—Nw/overweight–obese—Ov-Ob) and their relationship to BMI in Italian adolescents. Methods: In total, 144 students (F = 72; M = 72) aged between 11 and 13 y.o. participated in this study. They were divided into two groups by gender and into two subgroups based on weight (Nw/Ov-Ob). Standing long jump, sit-and-reach, 10 × 5 m shuttle and sit-up tests to assess MP were carried out, and an ad hoc questionnaire to evaluate PSP was administrated. Results: In comparing the Nw and Ov-Ob subgroups on the basis of BMI for each gender, statistically significant differences emerged in all motor skill tests administered. Regarding PSP, the Nw subgroup showed high and positive levels, while Ov-Ob showed low and negative levels, highlighting a relationship between high BMI and poor motor performance as well as negative psychophysical perception in the Ov-Ob subgroup. Conclusions: this study supports the importance of physical sport activity to regulate excess weight but also contribute to better psychophysical well-being.

## 1. Introduction

The World Health Organization (WHO) has reported an exponential increase in the number of obese children and adolescents, making effective preventive and therapeutic actions necessary [1]. Sedentary lifestyles, overweight and obesity in children and adolescents are current issues of great relevance [2], as these conditions can influence youth physical performance, health and psychophysical well-being [3,4] and are predictive factors for obesity in adulthood [5]; these are, consequently, relevant issues for public health. In an individual, a sedentary lifestyle will generate, as a consequence, hypotonia in the skeletal muscle system, with alterations in the cardio-respiratory system and the metabolic system [6,7], but can also affect self-esteem and socio-relational aspects [8]; furthermore, the presence of a high percentage of fat in overweight and obese subjects implies faster fatigue even in physical activities requiring little muscular effort [9], such as walking, running and climbing stairs, thus making children less skilled [10]. Additionally, obesity is associated with an increased risk of psychosocial problems such as low self-esteem and depression [11]: aspects that are not always taken into proper consideration.

During childhood and adolescence, the development of motor skills [12] is dependent on and can be stimulated by the experience undertaken and the movement opportunities offered, both onsite and online [13], in sports clubs, in schools and during free time. Kalogiannis [14] demonstrated both a positive correlation between participation in physical and sporting activity programs and the perception of the personal body and a correspondence between low levels of physical efficiency and the perception of competence in children and youth, highlighting the benefits of specific activity interventions on physical, psychological and socio-affective development. Cardel et al. [15] have examined the results of interventions based on behavioral, pharmacological, surgical and device interventions for obesity, reporting that these practices have not been effective at reducing adolescent obesity at the population level. A meta-analysis carried out by Sobol-Goldberg et al. [16] concerning school-based obesity prevention programs indicates that more recent studies have shown convincing evidence that school-based prevention interventions are at least mildly effective in reducing BMI in children, possibly because these newer studies tend to be longer and more comprehensive and include parental support. A systematic review carried out by Yuksel et al. [17], which examined the effectiveness of school-based preventive programs by including variables such as content, type and duration of physical activity, which can directly affect the success of the school-based programs, reports that the quality, duration and priority of physical activity intervention in comprehensive school-based programs and teacher capacity are some of the most important factors for preventing obesity and that if more impact is desired in school-based intervention programs, the focus of the programs should be physical activity and, as much as possible, physical activities should be implemented directly.

Physical and movement experience can provide various opportunities for growth, in which participants are fully involved not only on a physical motor sports level but also on cognitive, emotional and social levels [18]. The regular practice of physical sport activity during childhood and adolescence has a dual function [19]; on one hand, it contributes to energy balance and weight control, and on the other hand, it produces beneficial effects on motor development, the musculoskeletal system, cardio-respiratory function and psychological well-being [20]. A limited or even absent opportunity of physical activities can negatively influence motor skills [21] and consequently motor sport performance and, not to be underestimated, can also have repercussions on social development and self-esteem [22]. Physical performance in obese children and adolescents is often compromised by a reduced ability to carry out physical sport activities that require coordination, balance, agility, strength and resistance [23,24], with possible repercussions on opportunities for participation in recreational, sporting and recreational activities; for sport drop-out due to burnout situations [25,26]; and the risk of strong reduction in socio-relational opportunities. On a psychological level [27], physical and sporting activities have proven to be effective tools for promoting psychological well-being among adolescents [28] and reducing stress [29]; furthermore, they can promote, in an inclusive way, social relationships and the development of interpersonal skills [30]: fundamental aspects during childhood and adolescence. Aggregation into social, cultural and sporting groups can increase a sense of belonging and awareness [31] and social inclusion and reduce the risk of isolation, which is often observed in young people with obesity [32]. However, despite the known benefits and Global Recommendations [33], physical activity may have remained relatively stable (but low) or declined slightly over the past 10 years, sedentary behavior (especially recreational screen time) may have increased or remained relatively stable [34] and sedentarism due to COVID-19 has increased the complexity of these problems [6]. In Italy, according to the 2022 Italian Health Behaviour in School-aged Children (HBSC) survey [35], carried out on 89.321 adolescents, alarming trends emerged because of the eating and physical activity habits of the Italian adolescents. Incorrect dietary behaviors and low practicing of physical activity are unfortunately prevalent, especially in Southern Italy and with lower socio-economic conditions, and they produce a worrying percentage of obese (19%) and inactive/partially active adolescents (only 7.5% of youth practice daily physical activity in Italy). It is therefore fundamental to better understand and intervene in the effects due to excess weight in childhood and adolescence.

Considering the influences that excess weight can have on physical, psychological and social health during adolescence and the need to design preventive interventions and implement effective strategies, taking into account the different psychophysical characteristics of each individual, the present study is exploratory of the psychophysical self-perception (PSP), body mass index (BMI) and motor performance (MP) levels in a group of Italian adolescents in the lower secondary school setting, and it aims to assess some aspects of motor performance and perceived psychophysical well-being, according to gender and weight differences, as well as their relationships with the body mass index. The choice to focus attention on adolescents was due to the awareness that in this developmental age, overweight or obesity is not a transitory state of weight gain but an indicator of a broader disorder that can include multiple factors (i.e., metabolic, behavioral) on which to work to prevent repercussions in adulthood.

## 2. Materials and Methods

### 2.1. Study Design

Descriptive research was carried out in a lower secondary school setting involving a group of Italian adolescents selected on the basis of their accessibility and availability. A non-probability/convenience sampling methodology was adopted.

Data were collected through an ad hoc questionnaire, anthropometric measurements and physical performance tests; statistical analysis was performed to analyze the collected data.

### 2.2. Participants

One hundred and forty-four students (F = 72; M = 72) aged between 11 and 13 years, attending lower secondary school in Naples, participated in this study. The participants were divided into two groups based on gender, males and females, and into two subgroups based on weight differences (normal weight—Nw—vs. overweight–obese—Ov-Ob), as shown in Table 1.

Data were collected during curricular physical education lessons by a group of graduates in sports science and physical education teachers. The sports science graduates had experience in youth training at sports clubs, while the physical education teachers had experience in the competitive sports field and had at least 10 years of teaching experience. All intervention providers (sports science graduates and teachers) received instructions on the procedure and administration of the performance tests.

### 2.3. Inclusion and Exclusion Criteria

All of those who expressed their willingness to participate were included in this study after obtaining informed consent from their parents. The exclusion criterion was the presence of unfavorable health conditions. Through this inclusive approach in the selection of participants, it was possible to acquire a large sample, thus allowing an in-depth and representative analysis of the relationships between the participants’ physical performance, psychophysical self-perception and BMIs.

### 2.4. Instruments

In order to collect the data, the following instruments were used:

1. An ad hoc questionnaire on self-perception of emotional and motivational states, with 10 multiple-choice questions and different answer options;
2. A portable stadiometer (Seca 213 version; Intermed, Italy);
3. A weighing scale with high-precision sensors (Healthkeep T107 version; Healthkeep, Basel, Switzerland);
4. The Cole Scale: an assessment tool that defines normal weight, overweight and obese status using BMI centile curves and adjusted cut-offs for age and sex [29];
5. The following physical performance tests: standing long jump (SLJ), using a long jump platform with a sandpit and a metric roll; sit and reach (SiR), using a box of a 55 cm length × 45 cm width and 40 cm height with a graduated scale in cm (0–50) on it; 10 × 5 m shuttle run (SR); and abdominal resistance strength (sit-up) using a chronometer for both tests.

### 2.5. Description of the Physical Performance Tests

SLJ: The participants placed their feet behind the line drawn on the long jump platform over the edge of the sandpit, crouched down and, using the arms and legs, jumped horizontally as far as possible, landing with both feet in the sandpit. The distance from the edge of the sandpit was measured and recorded to the nearest impression made by each participant in the sandpit.

SiR: Each participant, from a sitting position with their legs fully extended, placed their bare feet on a box with a graduated plate on top (cm) and slowly flexed the torso forward, with the knees locked, trying to reach, with the fingers, as far away as possible on the scale plate. Once they reached the maximum point of flexion, they maintained the position for at least 2 s. The distance reached by each participant’s fingertips (cm) was measured and recorded.

SR: In the gym, two lines were drawn 5 m apart from each other. The participants stood with both feet behind the starting line. At the starting signal, they ran, as fast as possible, to the other line, they crossed it with both feet and then they changed direction 10 times. To complete the test, the participants had to run 50 m and make 9 changes of direction. The time taken was measured and recorded at the end.

Sit-Up: Each participant, in a supine position, with knees bent (90°), feet flat on the floor, arms across the chest and plantar support on the ground, had to perform as many torso elevations as possible in 30 s. When the timing started, the back was lowered so the shoulder blades touched the floor; then, the participant returned to the upward position. The total number of correct sit-ups in 30 s was measured and recorded.

### 2.6. Procedures

In the initial phase of this study, anthropometric data on the participants’ height and weight were collected to calculate their BMIs. Height was measured while standing and without shoes using the portable stadiometer, while weight was measured using weight scales with high-precision sensors and with the participants wearing light clothing. Using the scale of Cole et al. [36], the BMI of each participant was calculated, classifying them as Nw or Ov-Ob according to the BMI overweight and obesity cut-off points (age- and sex-specific) from 11 to 13 years old (Table 2).

The choice to consider BMI as a relevant variable arose from the importance it has to the health of young people in the developmental age, as highlighted in the literature [37]. It can perform a dual function: first, through its periodic detection, it can provide motivational feedback to children; furthermore, as a monitoring tool, it can provide the level of effectiveness of the proposed physical sport activity interventions.

All participants performed 4 physical performance tests, chosen for being practical, time-efficient and low in cost and equipment requirements and because of their validity/reliability in verifying physical performance in adolescents. The standing long jump (SLJ) provides a general index of muscular fitness in youth [37]; sit and reach (SiR) has a moderate mean criterion-related validity for estimating hamstring extensibility [38] and the 10 × 5 m shuttle run (SR) and abdominal resistance strength (sit-up) contribute to measuring the health-related components of fitness [39,40]. Each test was performed following standardized protocols to ensure consistency and reliability in the results. The data collected were carefully recorded and analyzed, providing a detailed picture of the motor skills of the subjects involved.

Finally, a questionnaire consisting of 10 questions was administered to acquire data about the self-perceptions of the emotional and motivational states of the participants.

### 2.7. Questionnaire

The questionnaire was administered to the participants in paper format, and accurate instructions were provided to ensure that the participants filled it in correctly. The participants had to indicate:

- General information (age, weight and height);
- If they practiced regular physical activity, choosing between 2 options (yes and no);
- The type and frequency of physical activity practiced and perceptions about: their psychophysical states, their self-confidence (item 5), feelings (items 6 and 8), anxiety and stress (item 8) and motivation (item 9) when practicing sport activities and how physical and sport activities influenced their social relationships (item 10). It was possible to choose between 5 options.

The combined use of the ad hoc questionnaire and physical performance tests provided a wider vision of the participants’ psychophysical levels.

### 2.8. Statistical Analysis

The data, reported in descriptive form, are expressed as mean and standard deviation (mean ± SD). After verifying the normality of the data distribution using the Shapiro–Wilk test (*p* > 0.05) and the homogeneity of the variances with the Levene test (*p* > 0.05), a *t*-test for independent samples was used to compare the performances between the subgroups (Nw and Ov-Ob) in the male group and the female group. For each item of the questionnaire, the participants’ responses were graphically reported in terms of frequency and percentage. To check the extent to which all the items of the questionnaire assessed the same characteristic, its internal consistency was calculated using Cronbach’s α and the associated 95% confidence intervals (CI). A Cronbach α of 1 indicated perfect reliability, with a cut-off of 0.70 indicating an acceptable internal consistency [41]. The non-parametric Chi-square test (χ^2^) was used in order to investigate the differences in perception between Nw and Ov-Ob in both the male and female groups. The significance level was set at *p* < 0.05.

All statistical tests were performed using IBM SPSS (version 22; IBM, Armonk, NY, USA).

## 3. Results

After checking the normality of the data with the Shapiro–Wilk test and the homogeneity of variances using Levene’s test, an independent samples *t*-test was performed to test for differences in the performance tests (SLJ, SiR, 10 × 5 m shuttle run, sit-up) between the subgroups (Nw and Ov-Ob) in both males and females. Cronbach’s alpha was calculated to assess the internal consistency of the ad-hoc questionnaire. The aim was to determine whether all items measured the same construct. Finally, the Chi-square test was used to assess differences in perception among participants.

Table 3 reports the results of the four physical performance tests (SLJ, SiR, SR, sit-up) performed by the participants, divided into the male and female groups and into the Nw and Ov-Ob subgroups. The results of these tests, including the calculation of BMI, clearly indicate that the subjects belonging to the Nw subgroup performed better than the subjects of the Ov-Ob subgroup, highlighting the relationship between performance and BMI.

Table 4 shows the comparisons between the female (Nw and Ov-Ob) and male (Nw and Ov-Ob) subgroups. The results of this comparison, through the independent samples *t*-test, provided a clear, significant difference for all the variables considered. To obtain the effect size, Cohen’s d was calculated.

The internal consistency of the questionnaire was good (Cronbach’s α coefficient [95% CI]: 0.86 [0.82–0.89]; *p* < 0.00). Table 5 shows the results of the questionnaire; the differences between the two subgroups are described in detail as absolute values and percentages. Based on the answers provided by the Nw subgroup, it is notable that they showed high and positive levels of self-perception about physical sport activity and their emotional and motivational states. Conversely, Ov-Ob showed low and negative levels of self-perception about physical sport activity and their emotional and motivational states. The Nw subgroup declared feeling confident or very confident in their physical abilities (75.6%); being happy or very happy when they participated in physical or sporting activities (86% and 94.2%); feeling anxiety or stress sometimes (74.4%) or rarely (19.9%); and being often (54.7%) or always (36%) motivated. Conversely, the Ov-Ob subgroup declared having low or very low confidence (72.4%); being neither sad nor happy (37.9%) or sad (48.3%) when they participated in physical or sporting activities; feeling anxiety or stress often (44.8%) or sometimes (37.9%); and being rarely (60.3%) or sometimes (25.8%) motivated to practice physical or sporting activities. Finally, about the influence of sport and physical activity on social relationships, 94.2% of the Nw subgroup declared that participation in physical or sporting activities helped them to make new friends (59.3%) and that they felt part of a group (34.9%), while the Ov-Ob subgroup declared that participation in physical or sporting activities did not have a great impact on relationships for 37.9%, but for 27.6%, it still helped them to create friendships and, for 20.7%, to feel part of a group. These results highlighted the relationship between psychophysical self-perception and BMI.

The results revealed a clear relationship between BMI, motor performance and psychophysical perception. The motor test results showed that the subjects with normal BMIs achieved significantly better performance compared with the Ov-Ob subjects, confirming that a higher BMI negatively affects motor performance, most likely due to reduced coordination and endurance. On the other hand, from the results of the psychophysical perception questionnaire, it emerged that the group of Nw subjects reported significantly higher levels of positive self-perception, motivation and psychophysical well-being compared with the group of Ov-Ob subjects. This confirmed the relationship between high BMI and poor motor performance, as well as negative psychophysical perception. The subjects with high BMIs tended to perform poorly on the motor tests and perceive themselves more negatively compared with the Nw subjects.

## 4. Discussion

This study aimed to explore psychophysical self-perception (PSP), body mass index (BMI) and motor performance (MP) levels in a group of Italian adolescents in the lower secondary school context through the assessment of some aspects of motor performance and perceived psychophysical well-being, according to gender and weight differences, as well as their relationships with the body mass index.

The data from the physical performance tests (Table 2) highlight that the Nw subgroups for each gender obtained better physical performance results than the Ov-Ob subgroups, just as those of the male group were better than the female one, except for the sit-and-reach test. Overall, this study highlights that when comparing the Nw and Ov-Ob subgroups on the basis of BMI, for each gender, statistically significant differences emerged in all physical performance tests administered (Table 3). This means that excess weight has a negative influence on motor skill performance, confirming that overweight/obese boys and girls have had poorer motor performance because they had deficiencies affecting both coordination and conditional abilities, such as reduced endurance, speed and/or strength in the lower limbs [10,42]. The BMI can influence motor skills, such as the ability to perform coordinated, purposeful movements, such as jumping and running, that require lifting one’s body weight. This overload can cause a reduction in physical efficiency and an increase in energy expenditure, with a consequently lowered performance and small quantity of physical activities. Children and adolescents with poor motor development may be less willing to participate in physical sport activities, resulting in a reduction in energy expenditure and an increased risk of excess weight [43]. The presence of overweight or obesity can also be associated with low self-esteem and a negative perception of one’s body [44], which leads to reduced participation in physical activities.

The questionnaire helped us to better understand the habits and perceptions of the participants. As regards the self-perception of psychophysical well-being, the results that emerged from the application of the Chi square to the answers given by the Nw and Ov-Ob subgroups to the questionnaire indicate statistically significant differences (*p* < 0.05) in all the comparisons carried out (*p* > 0.05), just as clear differences emerged in percentage terms. The questionnaire, in addition to investigating some aspects of self-perception, highlighted the participants’ habits in terms of quantity and type of physical activity carried out. In comparing the Nw and Ov-Ob subgroups’ emerged statistically significant differences, the percentages are evident in showing how the two subgroups are diametrically opposed, as 88.3% of the Nw subgroup regularly practiced a physical or sporting activity while 89.6% of the Ov-Ob subgroup did not practice regularly; furthermore, 88.4% of the Nw subgroup practiced a physical or sporting activity from 1 to 4 times a week, while 89.6% of the Ov-Ob subgroup did not or rarely practiced physical or sporting activity.

The evidence of this study confirmed other, previous studies on adolescents in Ov-Ob conditions [45,46], adding other factors that should not be underestimated or overlooked but about which adolescents should be educated, such as the emotional, motivational and relational implications that can influence BMI or vice versa.

What impressed a lot, in addition to the alarming trend of excess weight of Italian adolescents, especially those who live in southern Italy [35], is that in a longitudinal retrospective of the various Health Behaviour in School-aged Children (HBSC) National surveys carried out over the last 20 years in Italy (for a total of six surveys from 2002 to 2022), the indicators referring to low levels of physical activity and movement did not change, despite the implementation and exploitation of curricular physical education in school already established with the “Buona scuola” Italian school reform (2015); moreover, although many local, regional and national projects have been implemented over the years in partnership with public and private bodies, including the Italian Olympic Committee (CONI) (“schools open to sport” since 2017 and “student championships” that promote school sports competitions, just to mention the most widespread), physical activity and physical education programs failed to positively influence the motor habits of children by increasing their levels of physical activity.

There is an epidemic physical inactivity trend that has now extended to the pediatric age, and at the same time, the need and urgency to plan and implement targeted and effective physical and movement education action has emerged, starting in schools, as schools are privileged places to intercept adolescents and their families and to promote educational programs that stimulate the specific learning area of physical education that, in the National Guidelines for the curriculum of the first cycle of education [47], refers to the thematic core of “health and well-being, safety and prevention”. Here, health is interpreted as a state of physical, mental and social well-being, the result of the best balance between individual responsibilities (intended as the ability to make decisions) and environmental opportunities (intended as knowledges, services and interventions).

## 5. Conclusions

A high BMI leads to various negative effects, including decreased motor skills and therefore motor performance, and also implications about the perception of one’s psychophysical state and well-being. A poor level of motor skills and a low perception of one’s psychophysical abilities can determine an influence on psychological perspectives and participation in physical and sporting activities. Understanding the relationships between motor performance, psychophysical variables and excess weight could help researchers, teachers, instructors and all adults who work with children and adolescents to develop increasingly effective intervention strategies that take into account the different psychophysical characteristics and needs of the subjects.

The promotion of lifestyles and environments that are good for health requires a global system approach involving all sectors, which, with their policies, would interact regarding the various determinants of health. In this sense, the training and education sector, through universities and schools, can influence personal factors (i.e., self-confidence, feelings, motivation, stress and anxiety management), behaviors (i.e., motor habits, physical activity) and social integration (interpersonal relationships, social networks), given the specific life and study conditions in the school setting (services, education, etc.). To promote health, wellness and fitness in young people, researchers should develop appropriate strategies that are consistent with individual needs, interests and skills. The increase in overweight and obesity is the result of multifaceted determinants and their complex interactions over time [2]. A school, via the curriculum, school ethos and community, is an ideal context for educating young people about the importance of physical activity and the value of achieving and/or maintaining health-related fitness standards [48,49] and building the necessary skills for long-term behavioral change [50]. A major advantage of school-based interventions is the facilitation to implement and monitor changes in the school environment to promote healthy behaviors, reaching many adolescents from all social classes. Many school-based intervention studies promoting physical activity and a healthy lifestyle have been performed over the last two decades, and in general, the results show [51] that despite how school-based interventions, including counseling and access to an after-school exercise program, are theoretically promising with public health potential, they were not effective in reducing BMI or key obesogenic behaviors, highlighting that interventions targeted at the individual level are not likely to be sufficient in addressing the adolescent obesity epidemic without changes in social norms and the environment. The schools can make these changes possible.

### Limitations and Future Research Directions

A limitation of this study could be represented both by the difficulty in identifying questions that included the greatest number of elements in common between the two subgroups, Nw and Ov-Ob, and by the size of the sample; obviously, a larger sample size would produce more solid results. The nature of these limitations can be a stimulus for future research, encouraging the adoption of broader or more diversified approaches in sample selection in the analysis of factors that could influence the physical sport activity and health of young people and the underlying reasons for the impact of BMI on mental health and delving deeper into the physiological and psychological mechanisms behind associations between BMI and poor exercise ability.

## Figures and Tables

**Table 1:** Anthropometric characteristics of the sample.

Gender	Total = 144	Subgroup	Age	Weight	Height	BMI
Male	44	Nw	11.90 ± 0.72	46.43 ± 8.91	1.57 ± 0.14	18.87 ± 1.78
	28	Ov-Ob	12.02 ± 0.85	59.14 ± 10.12	1.56 ± 0.18	24.33 ± 2.64
Female	42	Nw	11.81 ± 0.78	45.43 ± 8.12	1.54 ± 0.15	19.16 ± 1.71
	30	Ov-Ob	11.93 ± 0.86	60.24 ± 10.92	1.55 ± 0.12	25.10 ± 2.88

Nw = normal weight; Ov-Ob = overweight–obese; BMI = body mass index.

**Table 2:** Overweight and obesity cut-off points from 11 to 13 years old according to the Cole Scale.

Gender	Age	Cut-Off Point, Overweight: kg/m^2^	Cut-Off Point, Obesity: kg/m^2^
Male	11	BMI = 20.55 to 25.09	BMI > 25.10
	11.5	BMI = 20.89 to 25.57	BMI > 25.58
	12	BMI = 21.22 to 26.01	BMI > 26.02
	11.5	BMI = 21.56 to 26.42	BMI > 26.43
	13	BMI = 21.91 to 26.83	BMI = 26.84
Female	11	BMI = 20.74 to 25.41	BMI > 25.42
	11.5	BMI = 21.20 to 26.04	BMI > 26.05
	12	BMI = 21.68 to 26.66	BMI > 26.67
	12.5	BMI = 22.14 to 27.23	BMI > 27.24
	13	BMI = 22.58 to 27.75	BMI > 27.76

**Table 3:** Results of physical performance tests.

Gender	N = 144	Subgroup	Sit-Up	Sit and Reach	SR (10 × 5)	SLJ	BMI
Male	44	Nw	16.20 ± 4.20	+6.00 ± 4.10	13.49 ± 1.47	1.50 ± 0.35	18.93 ± 1.78
	28	Ov-Ob	11.40 ± 4.50	+1.50 ± 4.40	15.22 ± 1.86	1.35 0.45	24.32 ± 2.64
Female	42	Nw	14.20 ± 4.40	+8.00 ± 3.20	13.95 ± 1.17	1.40 ± 0.32	19.10 ± 1.71
	30	Ov-Ob	10.10 ± 3.20	+2.50 ± 3.50	16.84 ± 1.06	1.24 ± 0.30	25.10 ± 2.88

N = number of subjects; sit-up = abdominal; sit and reach = torso flexion; SR = shuttle run 10 × 5; SLJ = standing long jump; BMI = body mass index; Nw = normal weight; Ov-Ob = overweight–obese.

**Table 4:** Independent samples *t*-test between females (Nw and Ov-Ob) and males (Nw and Ov-Ob).

Female	t	Df	*p*-Value	Cohen’s d
SLJ	2.412	70	0.027	0.51
sit-up	4.034	70	<0.001	0.60
SR (10 × 5)	−6.425	70	<0.001	0.78
sit and reach	6.051	70	<0.001	0.74
BMI	−8.996	70	<0.001	1.00
Male				
SLJ	2.537	70	<0.021	0.56
sit-up	4.330	70	<0.001	0.62
SR (10 × 5)	−3.945	70	<0.001	0.60
sit and reach	4.928	70	<0.001	0.66
BMI	−9.591	70	<0.001	1.02

SLJ = standing long jump; sit-up = abdominal; sit and reach = torso flexion; SR = shuttle run; BMI = body mass index; *p*-value = < 0.05. Cohen’s d (effect size) = 0.2–0.5, small; 0.5–0.8, moderate; and >0.8, large.

**Table 5:** Answers of psychophysical self-perception of participants.

1. General information	Age: 11–12–13 Height: ____ Weight: ____	**Subgroups**				**χ ²** ***p*-Value**	**Cramer’s V**
		**Nw**		**Ov-Ob**			
2. Do you regularly practice physical activity or sports?	Yes	76	88.3%	6	10.4%	0.0001	
	No	10	11.7%	52	89.6%		0.3
3. If yes to question 2, which physical or sporting activity(s) do you practice?	Swim	6	7.8%	0	0%	0.001	0.2
	Dance	10	13.2%	0	0%		
	Soccer	23	30.3%	0	0%		
	Basketball	12	15.8%	0	0%		
	Other	25	32.9%	6	10.4%		
4. How many times a week do you practice physical or sporting activity?	Never	0	0%	36	62.1%	0.0001	
	Rarely	10	11.6%	16	27.5%		
	1–2 times	54	62.8%	6	10.4%		0.4
	3–4 times	22	25.6%	0	0%		
	5 or more	0	0%	0	0%		
5. How confident do you feel in your physical abilities during physical activity or sports?	High	12	13.9%	0	0%	0.0001	
	Enough	53	61.7%	0	0%		
	I don’t know	21	24.4%	16	27.6%		0.4
	Low	0	0%	34	58.6%		
	Very low	0	0%	8	13.8%		
6. How happy do you feel emotionally when you participate in a physical activity or sport?	High	28	32.5%	0	0%	0.0001	
	Enough	46	53.5%	6	10.3%		
	I don’t know	12	14.0%	22	37.9%		0.4
	Low	0	0%	28	48.3%		
	Very low	0	0%	2	3.5%		
7. Do you ever feel anxiety or stress before or during physical activity or sports?	Always	0	0%	4	6.9%	0.0001	
	Often	5	5.8%	26	44.8%		
	Sometimes	64	74.4%	22	37.9%		0.3
	Rarely	17	19.8%	6	10.4%		
	Never	0	0%	0	0%		
8. How do you feel emotionally after doing physical or sporting activity?	Very happy	21	24.4%	0	0%	0.0001	
	Happy	60	69.8%	11	19.0%		
	Indifferent	5	5.8%	33	56.9%		0.4
	Sad	0	0%	14	24.1%		
	Very sad	0	0%	0	0%		
9. Do you feel motivated to practice physical activity or sports?	Always	31	36.0%	0	0%	0.001	
	Often	47	54.7%	0	0%		
	Sometimes	8	9.3%	15	25.8%		0.2
	Rarely	0	0%	35	60.3%		
	Never	0	0%	8	13.8%		
10. How has physical activity or sports influenced your social relationships? (You can select 2 answers.)	It helped me make new friends	102	59.3%	32	27.6%	0.031	0.2
	Participating makes me feel part of a group	60	34.9%	24	20.7%		
	It didn’t have a big impact	9	5.2%	44	37.9%		
	Participating doesn’t make me feel part of a group	0	0%	10	8.6%		
	It made my relationships with others worse	0	0%	6	5.2%		

## Data Availability

The original contributions presented in this study are included in the article; further inquiries can be directed to the corresponding authors.
